# A novel camera trapping method for individually identifying pumas by facial features

**DOI:** 10.1002/ece3.8536

**Published:** 2022-01-13

**Authors:** Peter D. Alexander, Derek J. Craighead

**Affiliations:** ^1^ Craighead Beringia South Kelly Wyoming USA

**Keywords:** camera traps, facial recognition, novel techniques, photo‐ID, population monitoring, *Puma concolor*, pumas, Wyoming

## Abstract

Camera traps (CTs), used in conjunction with capture–mark–recapture analyses (CMR; photo‐CMR), are a valuable tool for estimating abundances of rare and elusive wildlife. However, a critical requirement of photo‐CMR is that individuals are identifiable in CT images (photo‐ID). Thus, photo‐CMR is generally limited to species with conspicuous pelage patterns (e.g., stripes or spots) using lateral‐view images from CTs stationed along travel paths. Pumas (*Puma concolor*) are an elusive species for which CTs are highly effective at collecting image data, but their suitability to photo‐ID is controversial due to their lack of pelage markings. For a wide range of taxa, facial features are useful for photo‐ID, but this method has generally been limited to images collected with traditional handheld cameras. Here, we evaluate the feasibility of using puma facial features for photo‐ID in a CT framework. We consider two issues: (1) the ability to capture puma facial images using CTs, and (2) whether facial images improve human ability to photo‐ID pumas. We tested a novel CT accessory that used light and sound to attract the attention of pumas, thereby collecting face images for use in photo‐ID. Face captures rates increased at CTs that included the accessory (*n* = 208, *χ* ^2^ = 43.23, *p* ≤ .001). To evaluate if puma faces improve photo‐ID, we measured the inter‐rater agreement of 5 independent assessments of photo‐ID for 16 of our puma face capture events. Agreement was moderate to good (Fleiss’ kappa = 0.54, 95% CI = 0.48–0.60), and was 92.90% greater than a previously published kappa using conventional CT methods. This study is the first time that such a technique has been used for photo‐ID, and we believe a promising demonstration of how photo‐ID may be feasible for an elusive but unmarked species.

## Graphical abstract

Pumas (*Puma concolor*) are a challenging species to individually identify in conventional camera trap images due to their lack of distinctive flank markings. Individual identity from facial features has been successful for other “unmarked” species, but has relied on handheld telephoto cameras, which are not suitable for elusive species such as pumas. We developed a camera trap accessory to elicit front‐on camera trap images of pumas and found that it increased the likelihood of capturing puma face images; we also found an improvement in inter‐rater agreement among experts assigning identity to pumas using face images.

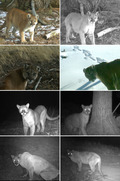

## 1 INTRODUCTION

Accurate estimates of abundance are fundamental to effective wildlife management and conservation. However, abundance estimates are often difficult to obtain, especially for rare or elusive species for which direct observation and live capture are difficult and costly (Gese, 2001; Kucera & Barret, 2011; McDonald, 2004). Motion‐triggered remote cameras, or camera traps (CTs), have thus become an important and often used tool for researching such species due to their ability to record wildlife observations autonomously, continuously, noninvasively, and at relatively low cost (Kucera & Barret, 2011). There are a wide variety of methods to estimate abundance using CT data, but those that incorporate individual detection histories in a capture–mark–recapture‐type framework (CMR, photo‐CMR; Karanth, 1995; Otis et al., 1978) are often considered the “gold standard” (Sollmann, 2018). CMR is not always feasible, however, due to the difficult nature of reliably identifying individuals in CT images (photo‐ID), especially for species that do not exhibit conspicuous and individually unique markings (e.g., stripes or spots). Many alternatives to CMR exist for such “unmarked” species (e.g., relative abundance indices, O’Brien et al., 2003; occupancy‐based abundance models, Royle & Nichols, 2003; spatial count models, Chandler & Andrew Royle, 2013; random encounter models, Rowcliffe et al., 2008), but carry important disadvantages, notably the inability to model individual capture heterogeneity which can be an important variable for elusive species with low detection rates (Harmsen et al., 2011; Sollmann, 2018). Spatial count models have furthermore been shown to produce imprecise estimates without auxiliary data which may be difficult to acquire for certain species (Sollmann et al., 2013), and random encounter models require a random CT placement strategy which may not be suitable or cost‐effective for elusive species that require targeted CT placement (Foster & Harmsen, 2012; Rowcliffe et al., 2013). Thus, those estimators which incorporate detection data at the individual level, including “partially marked” methods (i.e., mark–resight, McClintock et al., 2009), are preferred whenever photo‐ID is available.

For species with conspicuous flank markings, photo‐ID is relatively straightforward using CTs placed along travel paths to photo‐capture the flank markings of species such as tigers (*Panthera tigris*; Karanth, 1995) and jaguars (*Panthera onca*; Silver et al., 2004). Because markings are typically laterally asymmetrical, CTs are often placed in pairs to capture both flanks, thus avoiding issues of partial identity (McClintock et al., 2013). Of course, only a subset of animal species exhibits such markings; based on a survey of 176 carnivore species by Ortolani and Caro (1996), over 60% exhibited uniform flank coloration. Some research has shown that uniformly pelaged species may nevertheless be individually identifiable by subtler characteristics such as scars, tail kinks, or slight color variations (Kelly et al., 2008; Murphy et al., 2018; Sarmento et al., 2009). However, this remains controversial, and other research has found significant issues with misidentification and consequently inaccurate population estimates (Alexander & Gese, 2018; Foster & Harmsen, 2012; Güthlin et al., 2014; Oliveira‐Santos et al., 2010).

Certain species, while lacking flank markings, may exhibit other markings that are not readily visible from a typical lateral‐view CT setup. For example, several carnivores exhibit ventral patches of contrasting fur which can be photocaptured using imaginative CT techniques, such as using suspended bait to encourage an animal to stand erect and expose the patch to a CT; examples include wolverines (*Gulo*; Magoun et al., 2011), American martens (*Martes americana*; Sirén et al., 2016), and Asian black bears (*Ursus thibetanus*; Ngoprasert et al., 2012). Facial features are another potentially useful identifier which has been used to photo‐ID a wide variety of species, including African lions (*Panthera leo*; Pennycuick & Rudnai, 1970), elephant seals (*Mirounga leonine*, Caiafa et al., 2005), bottlenose dolphins (*Tursops truncates*; Genov et al., 2018), brown bears (*Ursus arctos*, Clapham et al., 2020), and several primate species (Deb et al., 2018). These studies employed a variety of identification methods, ranging from simple human judgment to modern machine learning techniques. However, all used images collected with conventional, handheld cameras—an impractical proposition for rare and elusive species for which CTs are especially advantageous. Facial‐ID has rarely been used in a CT framework, and only for species with distinctive flank markings. For example, identification of snow leopards (*Panthera uncia*) by Alexander et al. (2015) included facial spots along with other flank markings captured by conventional CTs. To our knowledge there has been no research into adapting CT methods explicitly to apply facial‐ID to an elusive species without natural markings.

Pumas (*Puma concolor*) are an elusive, sparsely occurring, and uniformly pelaged species for which population monitoring has proven challenging. Yet, due to the species’ huge distribution, ecological role as a top‐down regulator (Ripple & Beschta, 2006; Ripple et al., 2014; Rominger, 2018), and societal role as an umbrella or flagship species (Beier, 2009), population estimates are often sought for regional management or broad conservation strategies. While CTs are highly effective at photocapturing pumas, and potentially a cost‐effective method for estimating population size, pumas’ lack of conspicuous markings makes them a controversial subject for photo‐ID (Alexander & Gese, 2018; Foster & Harmsen, 2012). Anecdotally, puma facial features (e.g., those visible in a front‐on view such as the shape or condition of the eyes, rhinarium, or pinnae) are useful for identifying individuals but are not well captured with conventional CT techniques.

Here, we evaluate the feasibility of using facial features to photo‐ID pumas in CT surveys. We focus on two aspects: (1) the ability to successfully capture puma facial features with CTs, and (2) whether facial images improve human ability to photo‐ID pumas. For the former, we designed a CT accessory to encourage pumas to face a CT front‐on, thereby generating facial images instead of conventional lateral images. We assessed the ability of the device to capture face images, and examined possible behavioral reactions (Meek et al., 2016), including changes in detection rates that could indicate trap response. We then used inter‐rater agreement of photo‐ID as a proxy for accuracy of photo‐ID when using puma facial images (Alexander & Gese, 2018; Kelly et al., 2008; Mackey et al., 2007; McCarthy et al., 2018; Oliveira‐Santos et al., 2010). We note that our goal was to assess the feasibility of this novel method, as opposed to generating an unbiased detection history large enough to estimate abundance.

## 2 STUDY AREA

We deployed CTs year‐round at sites in the Jackson Hole basin in the Greater Yellowstone Area, in northwest Wyoming. All sites were on lands administered by the Bridger‐Teton National Forest. Elevations ranged from 2100 m to 3000 m. The climate was characterized by long snowy winters and short mild summers. The area was previously home to a long‐term puma radio‐collaring study; however, all collars were removed before the start of our study. Besides pumas, the study area included grizzly bears (*Ursus arctos*), black bears (*Ursus americanus*), and grey wolves (*Canis lupus*). Commonly occurring mesocarnivores included coyotes (*Canis latrans*) and red foxes (*Vulpes vulpes*). Primary prey species for puma included mule deer (*Odocoileus hemionus*), elk (*Cervus elaphus*), moose (*Alces alces*), bighorn sheep (*Ovis canadensis*), and various smaller mammals (Elbroch et al., 2013).

## 3 METHODS

**FIGURE 1:**
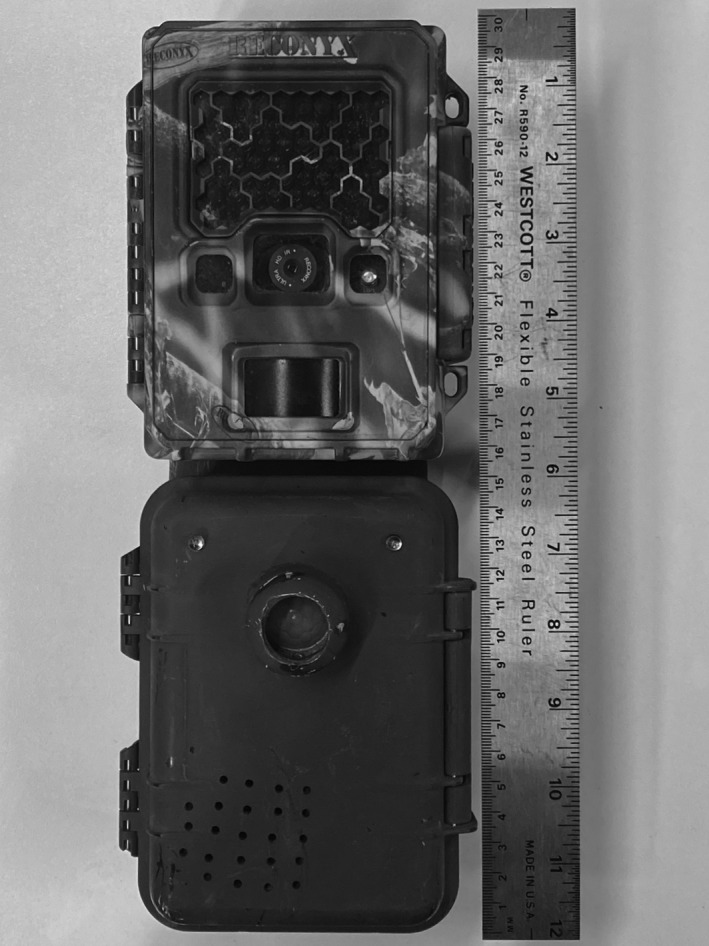
A standard camera trap with an “Attention caller device” (ACD). The camera and ACD were deployed together, and both triggered by motion. When activated, the ACD emitted light and sound to attract the attention of a puma, resulting in direct images of the puma's face. Camera traps and ACDs were deployed 2017–2020 in northwest Wyoming

We deployed two types of CT sites in the study area from mid‐2017 through 2019: conventional CTs and CTs that included a motion‐activated “attention caller” device (ACD), which we designed to operate alongside a CT. When triggered, the ACD played an audio recording of a juvenile puma call and illuminated a pair of light‐emitting diodes to elicit a curiosity response from the target animal. The ACD was enclosed in a weather‐proof casing, and was similar in size, battery life, and motion activation (i.e., used a passive integrated resistor to sense infrared radiation from animals) to a CT (Figure 1). It operated using the open‐source Arduino^®^ microcontroller platform. The materials used to build the ACD were relatively inexpensive, with each unit costing approximately US$33, with approximately half of that cost going toward the waterproof casing.

Sites were chosen based on likely puma travel routes or at puma community scrape sites (see Allen et al., 2014); approximately half were chosen opportunistically to include an ACD. The mean distance between sites was 12.99 km (SD = 9.52, min = 0.03, max = 54.42). Sites were moved after ~45 days if they failed to detect pumas. Approximately 6–12 sites were active at any given time during the survey. We fixed CTs to trees and mounted ACDs immediately above or below. We included a scent lure (e.g., Wildcat Lure No. 2 (Hawbaker & Sons, Fort Loudon, PA) or similar) at all sites. Lure was placed on the ground (~3–7 m) in front of cameras to encourage the animal to position itself at the center of the field of view and increase the likelihood of capturing useful images (Alexander & Gese, 2018; McBride & Sensor, 2015). We typically performed site visits to download CTs and refresh scent lure monthly; this period was longer at some sites due to wintertime access restrictions. We used Reconyx CTs (Hyperfire, Hyperfire2, or Ultrafire video models; Reconyx, Inc., Holmen, WI) programmed to take bursts of five photos with the “rapidfire” setting, or set to video when available. All CTs used infrared (IR) flash for low‐light conditions; daytime images were color and IR flash images were monochrome. We programmed ACDs with a 2‐second delay to allow an animal to fully enter the field of view before activating. Puma images were organized into detection events and were regarded as independent if greater than 30 minutes passed between photos from the same site.

### 3.1 Effects of the ACD

**FIGURE 2:**
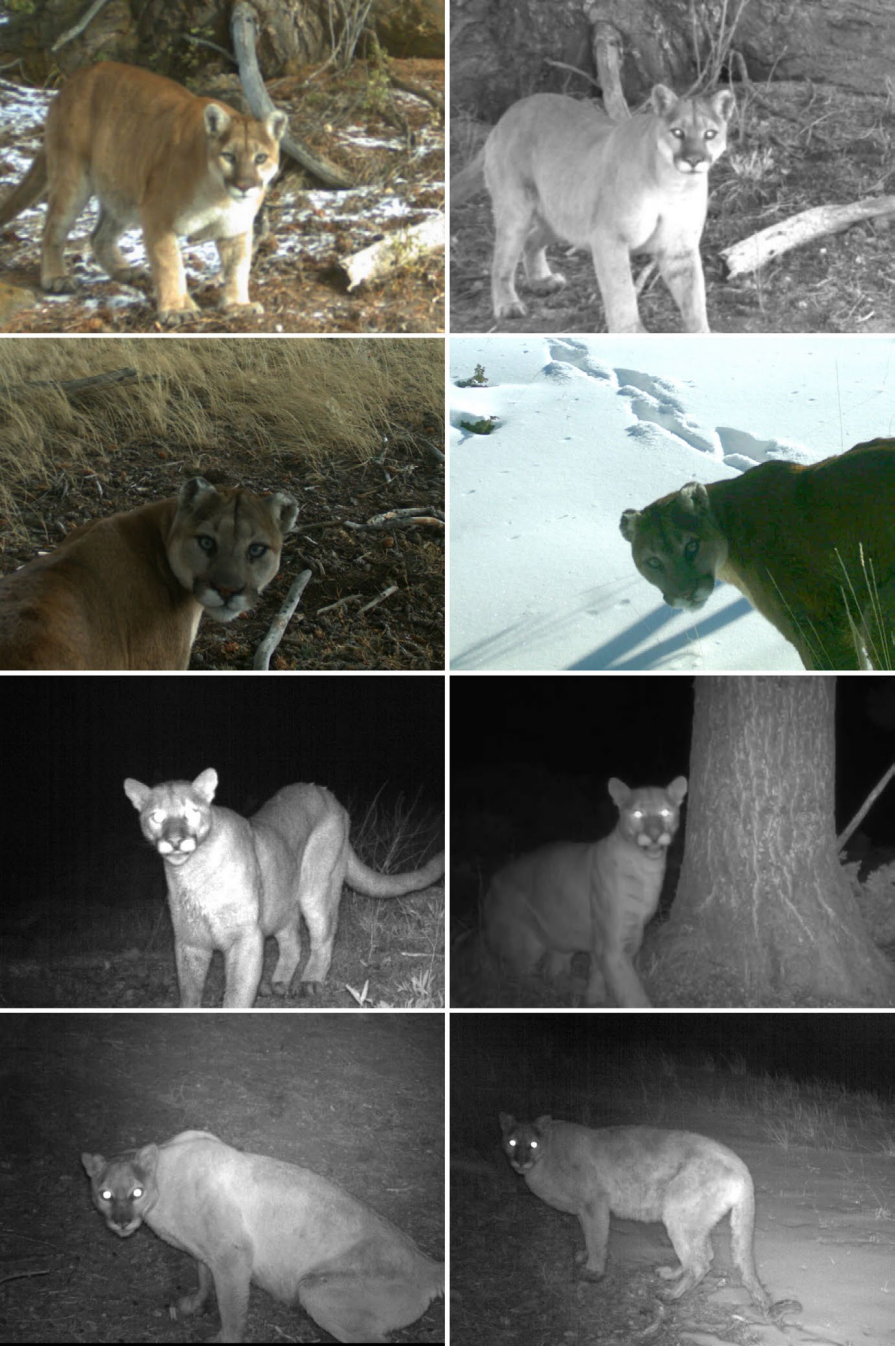
Camera trap photos of pumas taken in northwest Wyoming 2017–2020, using a novel method designed to capture pumas front‐on for facial‐ID. The photos are arranged by individual identity, with each row corresponding to an ID that had 100% agreement between five independent raters assigning photo‐ID to the image set

All analyses were completed using R (R Core Team, 2019). We categorized detection events as having successfully captured a complete image of the puma's face or not based on all facial features being visible in any single image within the event (Figure 2). We also grouped detections into four behavioral categories based on apparent behavior in images: (1) pumas moving away from the CT site in a manner that suggested fleeing (e.g., with a sudden change in speed or direction), (2) looking at the ACD with no other changes in behavior, (3) a strong curiosity response indicated by moving toward the CT to investigate, or (4) no apparent reaction. Note that face captures were not limited to the second category; fleeing and curiosity responses may have included face captures as well. We used Chi‐squared tests of independence to compare counts of detection events, face captures, and behavioral reactions between ACD sites and conventional CT sites, including whether events occurred during night or day (defined by astronomical twilight). To test for evidence that pumas learned to avoid ACD sites over time, we used the R package glmmTMB (Brooks et al., 2017) to build a Poisson generalized linear mixed model (GLMM) with daily count of detection events as the response variable (Henrich et al., 2020). We included CT site, season, and year as random effects. Explanatory variables included days since CT deployment, days since scent lure placement (both scaled), and two binary factors specifying whether the CT site included an ACD and whether the site was used by pumas as a community scrape site (based on presence of scrapes at deployment). All interactions were considered. We performed model selection using the buildmer package (Voeten, 2021), which finds the optimal model based on stepwise backward elimination using change in log‐likelihood as the criteria. We then compared similar models using Akaike's information criterion (AIC; Burnhan & Anderson, 2002), and diagnostic testing for overdispersion and zero inflation with the R package performance (Lüdecke et al., 2021b). We used type III analysis of variance (ANOVA) to assess significance of the predictor variables and their potential interactive effects by refitting the model with sum contrast coding for factors (Fox & Weisberg, 2018). Plots were created using the package sjPlot (Lüdecke, 2021a).

### 3.2 Photo‐ID analysis

**FIGURE 3:**
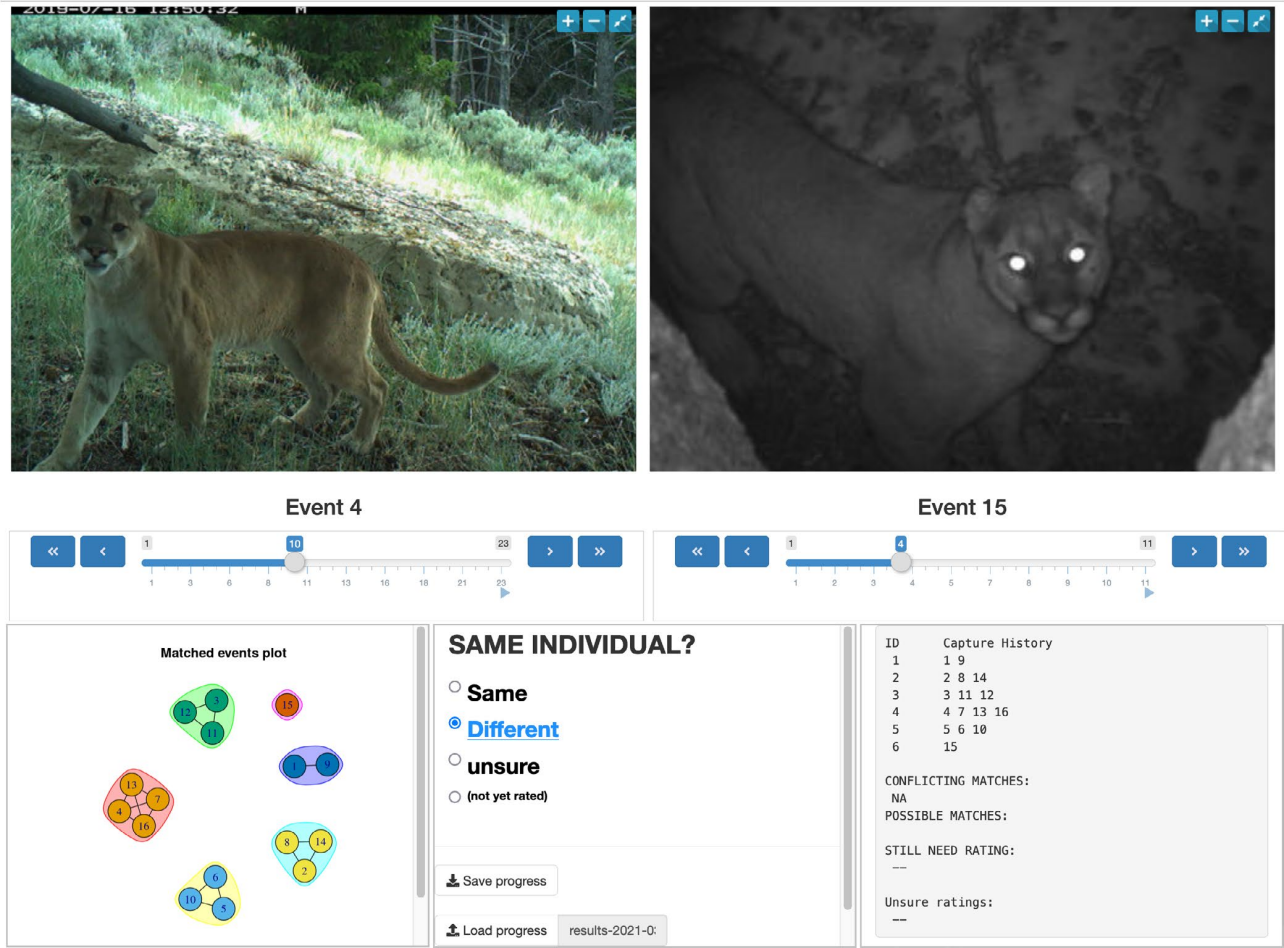
A screen capture of our web‐based application for matching pumas in camera trap images by individual identification. Each side of the screen displayed a camera trapping event; users were asked to classify each event pairing as being the same individual or not. As event pairs were matched, a graphical representation of puma IDs (lower left) was updated. Camera trap images were collected from 2017 to 2020 in northwest Wyoming

To evaluate the feasibility of using puma faces for photo‐ID, we measured the inter‐rater agreement between independent investigators assigning photo‐ID to detection events containing facial images; this process was similar to Alexander and Gese (2018). We developed an online application for assigning ID to images using the R package shiny (Chang et al., 2021). Investigators were presented detection events in a pairwise manner, with one event displayed on each side of the screen (Figure 3). Investigators could cycle through events (and the constituent photos) on each side of the screen independently. Investigators compared the events and rated each pairing as “same” or “different” based on perceived ID. All events included, but were not limited to, facial images. As investigators rated pairings, a network plot of events was updated to display the resulting identities (i.e., events were graphically grouped by ID based on the pairings rated as “same”) using the R package igraph (Csardi & Nepusz, 2006). The total number of event pairings was equal to $\left(\begin{array}{c} n\\ 2 \end{array}\right)$, where *n* was the number of events. Consequently, the number of pairings to evaluate increased exponentially with increased events; to avoid an onerous number of pairings to rate, we limited the number of events to 16, resulting in 120 pairings. The 16 events were randomly selected from those that included ≥1 face images. All participants had either professional or academic experience with camera trapping pumas. Agreement was measured with Fleiss’ kappa (Fleiss, 1971) using the R package rel (LoMartire, 2020). We calculated 95% confidence intervals following Fleiss et al. (1979). As with Alexander and Gese (2018), the true ID of pumas was unknown, and although high inter‐rater agreement would not necessarily indicate photo‐ID accuracy, low agreement would strongly suggest high potential for misidentification (Oliveira‐Santos et al., 2010). To further examine human ability to photo‐ID pumas using this method, we looked at several factors which may have affected agreement. We chose four binary attributes: (1) image type (monochrome vs. color), (2) ambient lighting (ambient lighting vs. CT flash only), (3) whether pumas exhibited clearly torn or missing pinnae, and (4) image resolution of puma faces. Resolution was defined by determining the number of pixels in the minimum bounding box for each detection's largest facial image; we then performed a median split. We used the above attributes to subset detection pairings in an either/or/both framework (e.g., only color pairings, only monochrome pairings, or pairings with one of each), and calculated Fleiss’ kappa for each. We used Spearman's rho to calculate the correlation between the attributes.

## 4 RESULTS

We recorded 13,375 days of CT effort at 61 sites. Of those days, 8,016 included an ACD. We collected 208 puma detection events, of which 98 were at ACD sites and 110 at conventional sites. Effort at community scrape sites totaled 7289 days, with 151 events occurring at those sites. Nighttime events made up 43.3% of detections and the IR flash was triggered in 81.7% of detections.

### 4.1 Effects of the ACD

**FIGURE 4:**
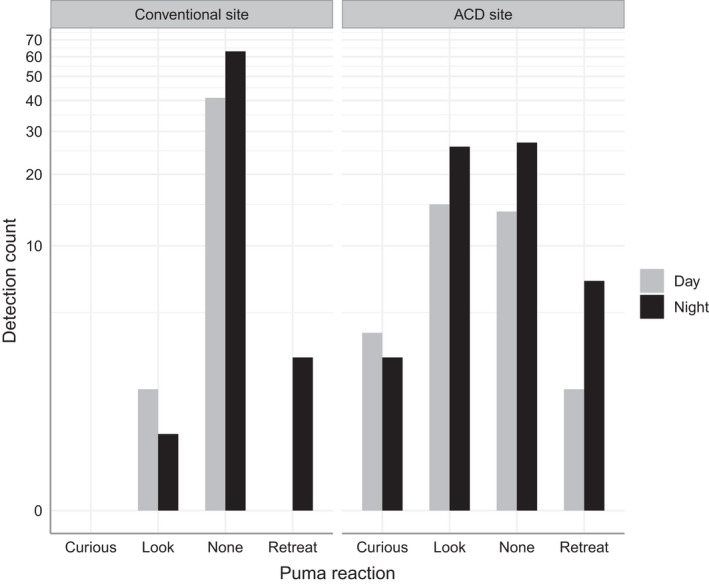
Counts of categorized puma behaviors at camera trap sites in Northwest Wyoming 2017–2020. Detection counts were grouped on whether the site included an “attention caller device” (ACD) used to elicit face images, and whether they occurred at night or day. The Y‐axis was log‐transformed. Categorizations were based on behavior apparent in camera trap images, and limited to (1) curiosity toward the camera, (2) looking at the camera without any other change in behavior, (3) no reaction at all, or (4) sudden retreat

Puma faces were captured in 52 of the ACD detection events (see Figure 3 for examples) and 12 of the conventional CT detection events, resulting in respective face capture rates of ~53.1% and ~10.9% per event (χ^2^ = 43.23, *p* ≤ .001). At CT sites without ACDs, ~94.5% of events did not have any apparent reaction; ACD site reactions were more varied, with ~83.7% categorized as either no reaction or a “look only” reaction (Figure 4). Retreat reactions occurred more at ACD sites than at conventional sites (χ^2^ = 3.98, *p* = .046). We found no significant differences between night and day events.

**FIGURE 5:**
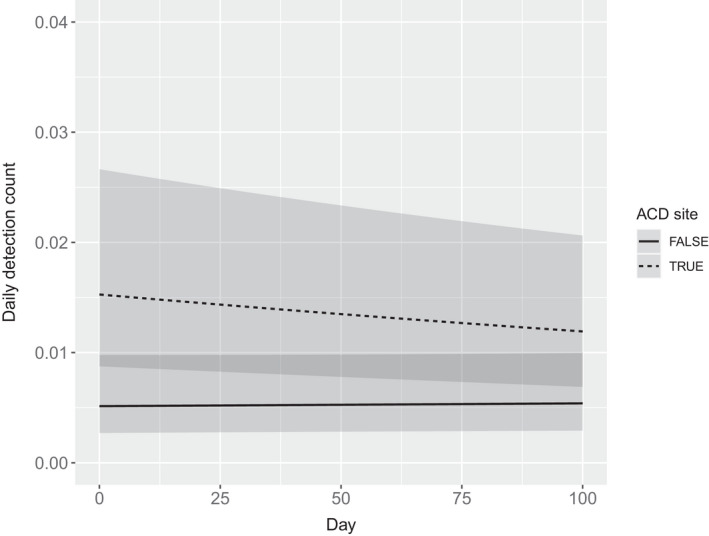
Predicted puma camera trapping rates using Poisson GLMM, from northwest Wyoming, 2017–2020. Regression lines indicate predictions for conventional cameras (solid line) and for cameras using an “attention caller device” (ACD; dotted line) to elicit frontal images of pumas. The shaded areas encompass the 95% confidence intervals

The top buildmer Poisson GLMM included 12 parameters and several interactive terms, including a three‐way interaction between ACD presence, scrape site status, and scent lure age. The second ranked model (ΔAIC = 2.0) was more parsimonious with eight parameters and was selected for our top model (Table 1). The interaction between scrape site status and ACD presence had a large positive effect on detection rates; the model also predicted a detection rate decrease of 0.163% per day (95% CI = 0.148–0.176) at all ACD sites, and an increase of 0.052% per day (95% CI = 0.009–0.102) when there was no ACD (Figure 5); we note all other candidate models predicted similar coefficients for these variables.

**TABLE 1:** Model coefficients, with standard errors and type III ANOVA measures of significance for parameters effecting daily counts of puma detections at camera trapping sites in northwest Wyoming, 2017–2020, including presence of an “attention caller device” (ACD), presence of puma scrapes at deployment, and number of days since the camera site was deployed

	Estimate	SE	χ^2^	*p*
(Intercept)	−5.34	0.39	386.62	≤.001
Scrape site	0.31	0.52	7.04	.008
ACD site	−0.18	0.48	1.57	.210
Days since deployment	0.094	0.17	2.80	.095
ACD * days since deployment	−0.58	0.19	9.79	.002
Scrape * ACD	1.24	0.69	3.23	.073

### 4.2 Photo‐ID analysis

We collected assessments of photo‐ID from five independent investigators. Of the 16 randomly sampled detection events, 10 were comprised of monochrome images with 8 of those occurring at night. Pumas exhibited conspicuous damage to one or both pinnae in six events; of those, a kinked tail was also visible in two detections. The median number of image pixels for puma faces was 39,216 (min = 13,338, max = 109,495). The mean number of identified individuals was 7.4 (*n* = 5, min = 6, max = 9, SD = 1.14). Nighttime events were (expectedly) correlated with events using IR flash (Spearman's rho = 0.78, *p* ≤ .001). We also found an unexpected correlation between puma face resolution and missing or damaged pinnae (Spearman's rho = 0.73, *p* ≤ .001).

**FIGURE 6:**
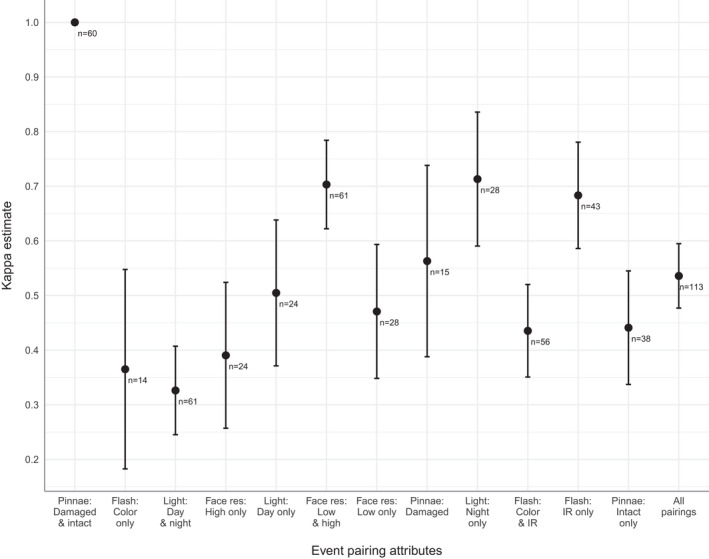
Estimates of agreement level (Fleiss’ kappa) between five independent investigators matching camera trap images of pumas by individual ID. Error bars indicate 95% confidence intervals. The different estimates represent agreement for various subsets of the image set based on ambient natural lighting (night vs. day/crepuscular images), use of infrared flash (color vs. monochrome), pixel resolution of the images, and whether pumas exhibited damaged pinnae (a particularly conspicuous and potentially distinguishing feature). Estimates were ordered from left to right by the sum of pairings that were positively matched by ID. The first estimate had perfect agreement and therefore no confidence intervals. Northwest Wyoming, 2017–2019

Fleiss’ kappa for the full dataset was 0.54 (95% CI = 0.48–0.60). Standard interpretations of this kappa would be categorized as “moderate” (Landis & Koch, 1977) or “intermediate to good” (Fleiss, 1971). This kappa was 92.90% greater than the kappa reported by Alexander and Gese (2018), which used conventional CT images of pumas and reported a “slight” or “poor” value of 0.18, with (P. Alexander and E. Gese, *unpublished data*) 95% confidence intervals of 0.14–0.23. In ~43.5% of the pairings with <100% agreement, a single investigator was in disagreement with the others. When these singletons were adjusted to match the consensus, Fleiss’ kappa increased to 0.76 (95% CI = 0.70–0.82), a “substantial” or “excellent” level of agreement. For the analysis of attribute subsets, most kappa estimates’ 95% confidence intervals overlapped those of the full dataset estimate (Figure 6). The lowest kappas were for pairings in which one or both events used color images. There was perfect agreement for event pairings in which one puma exhibited pinnae damage and the other did not; these pairings were predictably all rated as “different.”

## 5 DISCUSSION

Based on a comparison of our results and those of Alexander and Gese (2018), inter‐rater agreement improves greatly when using face images of pumas over conventional CT images for photo‐ID. We note that our study included fewer investigators (5 vs. 7); while this can raise the possibility of chance agreement, that issue is minimized in kappa statistics versus simple percent agreement (Gwet, 2010), and the 95% confidence intervals of the two kappas did not overlap. Our study also included a greater number of pairings to compare (120 vs. 105), and, unlike the 2018 study, investigators were not provided the advantage of knowing spatial/temporal distances between pairings (although they could likely infer which events were from the exact same site). The “substantial” agreement when using the adjusted ratings (i.e., switching the single investigator disagreements) was noteworthy; a similar reanalysis of the 2018 ratings resulted in a “moderate” agreement kappa of 0.48 (95% CI = 0.44–0.53; P. Alexander, *unpublished data*), which was still below our unadjusted kappa. These adjustments should be interpreted cautiously as they resulted in contradictory ID matchings which would need to be reconciled if used in a detection history.

Unsurprisingly, when one of the two events in a pairing exhibited visibly damaged pinnae, a “different” rating was always ascribed, resulting in the highest level of agreement for the subset analysis. This suggested that clear images of both pinnae are useful for distinguishing individuals, highlighting the value of frontal images of pumas as opposed to lateral, which, especially when only a single CT is used, result in lateral occlusion and partial identity issues (McClintock et al., 2013). It is also noteworthy that this feature was more correlated with kappa estimates than the image‐related features such as lighting. Notably, we did not see significant improvement in agreement when both events had distinctive pinnae, although the sample size of such pairings was relatively low with wide confidence intervals. The importance of this feature was also confounded by the almost certainly spurious correlation between image resolution and damaged pinnae, although kappa differences were comparatively low when pairings were based on resolution. We note missing pinnae were likely the result of frostbite, suggesting that (1) populations in warmer region may have lower frequencies of this feature, and (2) consideration should be given to the permanence of this feature in the context of a long‐term survey. Indeed, this issue may have affected our results due to the timespan of our detection set, and the importance of minimizing survey length should be emphasized (Kelly et al., 2008). Surprisingly, we found the monochrome pairings subset had increased estimates of kappa, and pairings with color images were lower. One possibility was that daytime images introduced varied angles of shadows as opposed to the uniformly directed lighting of the IR flash. Of course, these results should be interpreted carefully due to the small sample sizes, as well as the possibility of latent correlations with the actual ID of pumas, which likely varied in their identifiability.

The ACDs significantly improved the success rate of capturing face images compared to the conventional CTs. While much lower than the raw puma detection rates, the face capture rate at ACD sites (~53%) was higher than a theoretical conventional flank capture rate, assuming single CTs and a 50% chance of capturing either the right or left flank. The only CT study incorporating facial‐ID that we are aware of is Alexander et al. (2015), which reported a 32% face capture rate of snow leopards using CTs placed in a manner to attempt to capture animals head‐on. Improvements in the ACD face capture rate should be attainable, possibly through greater number of CTs per station, or an improved field of view for the CTs. Indeed, we did not count some face detections due to the puma being partially out of frame despite apparently eliciting the desired response to the ACD.

Clearly, the ACD reduced an element of non‐invasiveness normally associated with CTs, since detections explicitly required a behavioral response. The proportion of retreat reactions, while low, did increase with the ACD. We note that we also recorded such reactions without the ACD, namely in nighttime detections when pumas were likely reacting to the infrared flash. Our detection rate GLMM predicted a low decrease in detections over time, possibly due to an avoidance behavior. However, the predicted decrease was small, with the 95% confidence intervals overlapping those of conventional CTs, and may not affect a typical CT survey lasting only a few months. Importantly, we also report overall greater detection rates at ACD sites when deployed at community scrape sites; it is possible that this reflects some interactive behavioral response, but it also may be due to our opportunistic sampling strategy which placed ACDs at sites with high probability of puma visitation.

In this study, we assessed human ability to photo‐ID pumas. However, it is worth noting the increasing role of machine learning techniques for photo‐ID, which may reduce human effort and bias. While photo‐ID using machine learning could theoretically use CT images of individuals from any angle, facial‐ID is a more tractable machine learning problem and has long been in use for human recognition; more recently, it has been extended to a variety of unmarked wildlife species (Clapham et al., 2020; Deb et al., 2018). Our development of the ACD is therefore pertinent, as it could operate, in effect, as an in situ feature extractor in a machine learning framework.

Camera traps have yielded important improvements in our ability to monitor rare and elusive species, especially in terms of cost‐effectiveness and noninvasiveness. However, there are many elusive but unmarked species for which the advantages of CTs stop short of providing reliable population estimates. Pumas are one of the more glaring examples of such a species, given their huge distribution and ecological importance, as well as the ostensible cost‐effectiveness of CTs to collect data on pumas. This work strove to address some of these limitations and hopefully will encourage further research into similar field and analytical methods.

## CONFLICTS OF INTEREST

The authors have no conflicts of interest to declare.

## AUTHOR CONTRIBUTIONS

**Peter D. Alexander:** Conceptualization (lead); Data curation (lead); Formal analysis (lead); Methodology (lead). **Derek J. Craighead:** Funding acquisition (equal); Project administration (lead); Writing – review & editing (supporting).

## Data Availability

The detection data and photo‐ID agreement data have been uploaded to Dryad at https://doi.org/10.5061/dryad.j3tx95xfc. The R code will be available from the author by request.
